# iTRAQ-based comparative proteomic analysis provides insights into somatic embryogenesis in *Gossypium hirsutum* L.

**DOI:** 10.1007/s11103-017-0681-x

**Published:** 2017-12-06

**Authors:** Hua-Guo Zhu, Wen-Han Cheng, Wen-Gang Tian, Yang-Jun Li, Feng Liu, Fei Xue, Qian-Hao Zhu, Yu-Qiang Sun, Jie Sun

**Affiliations:** 1 College of Agriculture/The Key Laboratory of Oasis Eco-Agriculture, Shihezi University, Shihezi, 832000 Xinjiang China; 2 CSIRO Agriculture and Food, GPO Box 1700, Canberra, ACT 2601 Australia; 3 College of Life Sciences, Key Laboratory of Plant Secondary Metabolism and Regulation of Zhejiang Province, Zhejiang Sci-Tech University, Hangzhou, 310018 Zhejiang China; 4 Jingchu University of Technology, Jingmen, 448000 Hubei China

**Keywords:** *Gossypium hirsutum* L., Proteomic, Somatic embryogenesis, Polyamines, Stress-response

## Abstract

**Key message:**

iTRAQ based proteomic identified key proteins and provided new insights into the molecular mechanisms underlying somatic embryogenesis in cotton.

**Abstract:**

Somatic embryogenesis, which involves cell dedifferentiation and redifferentiation, has been used as a model system for understanding molecular events of plant embryo development in vitro. In this study, we performed comparative proteomics analysis using samples of non-embryogenic callus (NEC), embryogenic callus (EC) and somatic embryo (SE) using the isobaric tags for relative and absolute quantitation (iTRAQ) technology. In total, 5892 proteins were identified amongst the three samples. The majority of these proteins (93.4%) were found to have catalytic activity, binding activity, transporter activity or structural molecular activity. Of these proteins, 1024 and 858 were differentially expressed in NEC versus EC and EC versus SE, respectively. Compared to NEC, EC had 452 and 572 down- and up-regulated proteins, respectively, and compared to EC, SE had 647 and 221 down- and up-regulated proteins, respectively. KEGG (Kyoto Encyclopedia of Genes and Genomes) analysis indicated that genetic information transmission, plant hormone transduction, glycolysis, fatty acid biosynthesis and metabolism, galactose metabolism were the top pathways involved in somatic embryogenesis. Our proteomics results not only confirmed our previous transcriptomic results on the role of the polyamine metabolic pathways and stress responses in cotton somatic embryogenesis, but identified key proteins important for cotton somatic embryogenesis and provided new insights into the molecular mechanisms underlying somatic embryogenesis in cotton.

## Introduction

Upland cotton (*Gossypium hirsutum* L.) is one of the most important economic crops, and the earliest commercialized transgenic crop worldwide. Above 70% cotton cultivars now used in China contains pest-resistant genes that were transformed by the *Agrobacterium*-mediated method (Zhang et al. 2015).

Somatic embryogenesis (SEM) not only provides a technique for gene transformation, but also serves as a model system for study of zygotic embryo development in plants (Yang and Zhang 2010; Zimmerman 1993). Transition from non-embryogenic calli (NEC) to embryogenic calli (EC), and from EC to somatic embryo (SE) are the two key steps of SEM. In cotton, plant regeneration via SEM occurs in only few genotypes, which leads to low efficiency in gene function study and development of new cultivars through *Agrobacterium*-mediated transformation.

Many studies have investigated physiological and biochemical changes during SEM in various plant species with a focus on understanding the mechanisms of gene regulation related to SEM. These efforts identified genes differentially expressed in somatic embryos, highlighted the pathways likely to be involved in SEM and discovered DNA or protein markers for SEM (Mantiri et al. 2008). *SOMATIC EMBRYOGENESIS RECEPTOR-LIKE KINASE* (*SERK*) from *Daucus carota* was the first identified marker gene with a crucial role in SEM (Schmidt et al. 1997). Subsequently, a number of genes differentially expressed during SEM that are related to auxin synthesis, transport, metabolism, signaling and stress responses were reported (Jiménez 2005; Jiménez and Thomas 2006; Jin et al. 2014; Thomas et al. 2002; Yang et al. 2012; Zeng et al. 2007). The roles of several genes in SEM have been well-characterized, including *WUSCHEL* (Zuo et al. 2002), *LEAFY COTYLEDON* (*LEC)* (Gaj et al. 2005), *Arabinogalactan protein 1* (*AGP1*) (Letarte et al. 2006), *Glutathione-S-transferase* (*GST*) (Singla et al. 2007), *SOMATIC EMBRYO RELATED FACTOR1* (*MtSERF1*) (Mantiri et al. 2008), *BABY BOOM (BBM*) (Kulinska-Lukaszek et al. 2012) and *Agamous-like 15* (*AGL15*) (Yang et al. 2014; Zheng et al. 2013). Recently, a global transcriptome analysis of *Arabidopsis* embryogenesis provided a foundation for future research on genetic and epigenetic control of plant embryogenesis (Wickramasuriya and Dunwell 2015). On the other hand, molecular marker technology has been applied to identify quantitative trait loci related to SEM in upland cotton (Xu et al. 2015; Zhang et al. 2011). Furthermore, proteomic profile analysis has also been applied to study molecular mechanism of SEM in some plants (Bian et al. 2010; Heringer et al. 2015; Sun et al. 2013; Tan et al. 2013; Vale et al. 2014; Zhao et al. 2015). In cotton, a comparison of proteome dynamics between globular and cotyledonary embryos indicated that stress response, hormone homeostasis, and respiration and photosynthesis determined somatic embryo differentiation (Ge et al. 2014). These studies described the expression changes of a large number of genes or proteins during SEM and provided abundant clues for elucidation of the molecular mechanisms underlying SEM.

In this study, the isobaric tags for relative and absolute quantitation (iTRAQ) approach was applied to study proteome changes and to identify differentially accumulated proteins (DAPs) amongst the three key SEM stages in cotton, including NEC, EC and SE. Through identification and annotation of DAPs, we uncovered the key genes/proteins and pathways involved in cotton SEM. The results generated in this study provide a valuable foundation for further investigation of the roles of DAPs in cotton SEM.

## Materials and methods

### Plant materials

Seeds of cotton cultivar Xinluzao 33 (*G. hirsutum* L.) were decoated and imbibed in 0.1% (w/v) HgCl_2_ for 10 min, then rinsed three times by sterile distilled water. Seeds were germinated aseptically in jam jars containing ½ MS medium (Murashige and Skoog 1962). Hypocotyl sections (6–8 mm) from 6 days-old seedlings were used as explants for callus induction. The explants were cultured in MSB supplemented with 0.1 mg/L 2,4-D and 0.1 mg/L Kinetin (KT), and sub-cultured monthly. Samples were collected from the following three stages of SEM (1) NEC from explants cultured in MSB for 45 days, (2) induced EC and (3) SEs including globular, heart, torpedo and cotyledonary embryos. The collected samples were immediately frozen in liquid nitrogen, and stored at − 80 °C before protein extraction. Each sample was repeated three times. Culture mediums used in this study was listed in our previously published paper (Cheng et al. 2015).

### Protein preparation

Samples were ground into powder in liquid nitrogen and extracted with lysis buffer (7 M urea, 2 M thiourea, 4% CHAPS, 40 mM Tris–HCl, pH 8.5) containing 1 mM PMSF and 2 mM (final concentration) EDTA. After 5 min, 10 mM (final concentration) DTT was added to the samples. The suspension was sonicated at 200 W for 15 min and then centrifuged at 30,000×*g* for 15 min at 4 °C. The supernatant was mixed well with 5× volume of chilled acetone containing 10% (v/v) TCA. After overnight incubation at − 20 °C, the samples were centrifuged at 30,000×*g* for 5 min at 4 °C, and the supernatant was discarded. The precipitate was washed three times with chilled acetone. After air-dry, the pellet was dissolved in lysis buffer (7 M urea, 2 M thiourea, 4% NP40, 20 mM Tris–HCl, pH 8.0–8.5). The suspension was sonicated at 200 W for 15 min and centrifuged at 30000×g for 15 min at 4 °C. The supernatant was then transferred to another tube. To reduce disulfide bonds in proteins, 10 mM (final concentration) DTT was added and incubated at 56 °C for 1 h. Subsequently, 55 mM (final concentration) IAM was added and incubated for 1 h in the darkroom to block cysteines. The supernatant was then mixed well with 5× volume of chilled acetone for 2 h at − 20 °C to precipitate proteins. After centrifugation at 30000×*g* for 5 min at 4 °C, the supernatant was discarded. The pellet was dissolved in 500 μL of 0.5 M TEAB (Applied Biosystems, Milan, Italy) after 5 min of air-dry, and sonicated at 200 W for 15 min. Finally, samples were centrifuged at 30,000×*g* for 15 min at 4 °C. The supernatant, i.e. protein solution, was transferred to a new tube, quantified and then kept at − 80 °C before further analysis.

### iTRAQ labeling and SCX fractionation

For each sample, 100 μg of total protein was digested by Trypsin Gold (Promega, Madison, WI, USA) at 37 °C for 16 h with a ratio of protein:trypsin = 30:1. After trypsin digestion, the solutions were dried by vacuum centrifugation. Peptides were then reconstituted in 0.5 M TEAB and processed using the 8-plex iTRAQ reagent kit according to the manufacture’s protocol (Applied Biosystems). Briefly, one unit of iTRAQ reagent was thawed and reconstituted in 24 μL isopropanol. Samples were labeled with the iTRAQ tags as follow: Sample NEC1/2 (114/116 tag), Sample EC1/2 (119/117 tag), Sample SE1/2 (118/121 tag). The isobaric tags labeled peptides were incubated at room temperature for 2 h, then pooled and dried by vacuum centrifugation.

SCX chromatography was performed with a LC-20AB HPLC Pump system (Shimadzu, Kyoto, Japan). The iTRAQ-labeled peptide mixtures were reconstituted with 4 mL buffer A (25 mM NaH_2_PO_4_ in 25% ACN, pH 2.7) and loaded onto a 4.6 × 250 mm Ultremex SCX column containing 5-μm particles (Phenomenex). The peptides were eluted at a flow rate of 1 mL/min with buffer A for 10 min, 5–60% buffer B (25 mM NaH_2_PO_4_, 1 M KCl in 25% ACN, pH 2.7) for 27 min, 60–100% buffer B for 1 min. The system was then maintained at 100% buffer B for 1 min before equilibrating with buffer A for 10 min prior to the next injection. Elution was monitored by measuring the absorbance at 214 nm, and fractions were collected every 1 min. The eluted peptides were pooled into 20 fractions, desalted with a Strata X C18 column (Phenomenex) and vacuum-dried.

### LC–ESI–MS/MS analysis based on Q EXACTIVE

Each fraction was resuspended in buffer C (2% ACN, 0.1% FA) and centrifuged at 20,000×*g* for 10 min, the final concentration of peptide was about 0.5 μg/μL on average. 10 μL supernatant was loaded on a LC-20AD nanoHPLC (Shimadzu, Kyoto, Japan) by the autosampler onto a 2 cm C18 trap column. Then, the peptides were eluted onto a 10 cm analytical C18 column (inner diameter 75 μm) packed in-house. The samples were loaded at 8 μL/min for 4 min, then the 44 min gradient was run at 300 nL/min starting from 2 to 35% buffer D (98% ACN, 0.1% FA), followed by 2 min linear gradient to 80%, and maintained at 80% buffer D for 4 min, and finally return to 5% in 1 min.

The peptides were subjected to nanoelectrospray ionization followed by tandem mass spectrometry (MS/MS) in a Q EXACTIVE (Thermo Fisher Scientific, San Jose, CA) coupled online to the HPLC. Intact peptides were detected in the Orbitrap at a resolution of 70,000. Peptides were selected for MS/MS using high-energy collision dissociation (HCD) operating mode with a normalized collision energy setting of 27.0; ion fragments were detected in the Orbitrap at a resolution of 17,500. A data-dependent procedure that alternated between one MS scan followed by 15 MS/MS scans was applied for the 15 most abundant precursor ions above a threshold ion count of 20,000 in the MS survey scan with a following Dynamic Exclusion duration of 15 s. The electrospray voltage applied was 1.6 kV. Automatic gain control (AGC) was used to optimize the spectra generated by the Orbitrap. The AGC target for full MS was 3e6 and 1e5 for MS2. For MS scans, the m/z scan range was 350–2000 Da. For MS2 scans, the m/z scan range was 100–1800.

### Data analysis

Raw data files acquired from the Orbitrap were converted into MGF files using Proteome Discoverer 1.2 (PD 1.2, Thermo), 5600 msconverter and the MGF file were searched. Protein identification was performed by using Mascot search engine (Matrix Science, London, UK; version 2.3.02) against the database.

For protein identification, a mass tolerance of 5 Da was permitted for intact peptide masses and 13 Da for fragmented ions, with allowance for one missed cleavages in the trypsin digests. Gln->pyro-Glu (N-term Q), oxidation (M), deamidated (NQ) as the potential variable modifications, and carbamidomethyl (C), iTRAQ8plex (N-term), iTRAQ8plex (K) as fixed modifications. The charge states of peptides were set to +2 and +3. Specifically, an automatic decoy database search was performed in Mascot by choosing the decoy checkbox in which a random sequence of database is generated and tested for raw spectra as well as the real database. To reduce the probability of false peptide identification, only peptides with significance scores (≥ 20) greater than “identity” at the 99% confidence interval by a Mascot probability analysis were counted. And each confident protein was identified with at least one unique peptide.

The quantitative protein ratios were weighted and normalized by the median ratio in Mascot. We only used ratios with p values < 0.05, and considered those with a fold change of > 1.5 as significant.

### Protein function annotation

Functional annotations of the proteins identified were conducted using the Blast2GO program against the non-redundant protein database (NR; NCBI). The KEGG database (http://www.genome.jp/kegg/) and the COG (Cluster of Orthologous Groups) database (http://www.ncbi.nlm.nih.gov/COG/) were used to classify and group the proteins. COG is the database for orthologous protein classification. Every protein in COG is supposed to derive from a same protein ancestor. KEGG pathway is a collection of manually drawn pathway maps representing our knowledge on the molecular interaction and reaction networks. Gene Ontology (GO) is an international standard of gene function classification, which provides a set of dynamic updating controlled vocabulary to describe attributes of genes and gene products. GO has three Ontologies describing molecular function, cellular component, and biological process.

### RNA extraction and quantitative real-time PCR

Total RNA was extracted from NEC, EC and SE of Xinluzao 33 using a modified cetyltrimethyl ammonium bromide (CTAB) method (Luo et al. 2003) and was stored at − 80 °C before use. The quality of total RNA was checked on a 1% (w/v) ethidium bromide-stained agarose gel. 1 µg aliquot of total RNA was used for the first-strand cDNA synthesis with the M-MLV reverse transcriptase (TaKaRa) following the manufacturer’s instructions. Quantitative real-time PCR (qRT-PCR) was performed to detect gene expression level. The gene-specific primers used in qRT-PCR are listed in Table 1, and the cotton EF1α gene was used as an internal control. The qRT-PCR assays were performed with SYBR Premix Ex Taq (TaKaRa) on an Mx3000p system (Agilent, USA). Each reaction (25 μL) contained 4 μM of each primer, 2 μL of cDNA (1:100 diluted), and 10 μL of PCR buffer for the Eva Green Master Mix. Thermal cycling conditions were pre-incubation at 95 °C for 2 min, followed by 40 cycles of 94 °C for 15 s, 56 °C for 20 s, and 72 °C for 20 s. Relative expression ratios of the target genes were calculated based on the standard equation (Bogs et al. 2005). The expression assay was performed using three independent biological replicates and each biological replicate was performed with three technical repeats.

**Table 1:** Primers used in qRT-PCR

Gene accession	Sense primer	Antisense primer
Unigene22318_All	5′-ATCACCGACACCTACTCCCT-3′	5′-GAACAGTTATCTCCCGACCA-3′
CL8598.Contig1_All	5′-GCGGTGATTGGTCTGAGATG-3′	5′-CGATGAGTTGTTGATTGGGTT-3′
CL12349.Contig2_All	5′-CATTACGGGTCGTTTAGCC-3′	5′-ATCTCCCAACTTGCATTCC-3′
CL543.Contig2_All	5′-TGACATGCTGCCTTCGTTT-3′	5′-CCCTGTATTGCTGTGACCC-3′
CL3039.Contig1_All	5′-GAGATCACCTGTGAAGCGAGAA-3′	5′-TCCCACTGTAATCCGAACCA-3′
CL2767.Contig2_All	5′-TCCAGCCTCAGCAACCTAC-3′	5′-GGTTTGAAGCCCAGGAGTG-3′

## Results and discussion

### Somatic embryogenesis in cotton *cv* Xinluzao 33

**Fig. 1:**
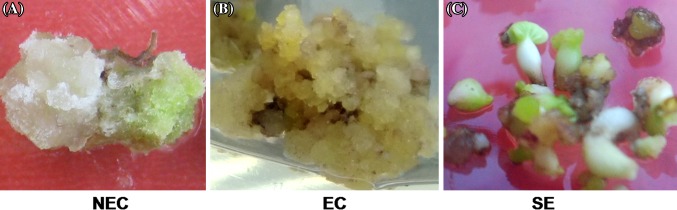
Samples used for proteomic assays. **a** 45-day non-embryogenic callus (NEC); **b** embryogenic callus (EC); **c** somatic embryos (SEs)

Embryogenic callus (EC) of Xinluzao 33 was formed from non-embryogenic callus (NEC) after about 6 months of culture. Differentiation of somatic embryos (SEs) from embryogenic callus was the most restrictive step during cotton SEM and plantlet regeneration. To identify proteins with a key role in cotton SEM, we sampled NEC, EC and SEs for protein preparation and iTRAQ analysis (Fig. 1a–c).

### Protein identification by iTRAQ

**Fig. 2:**
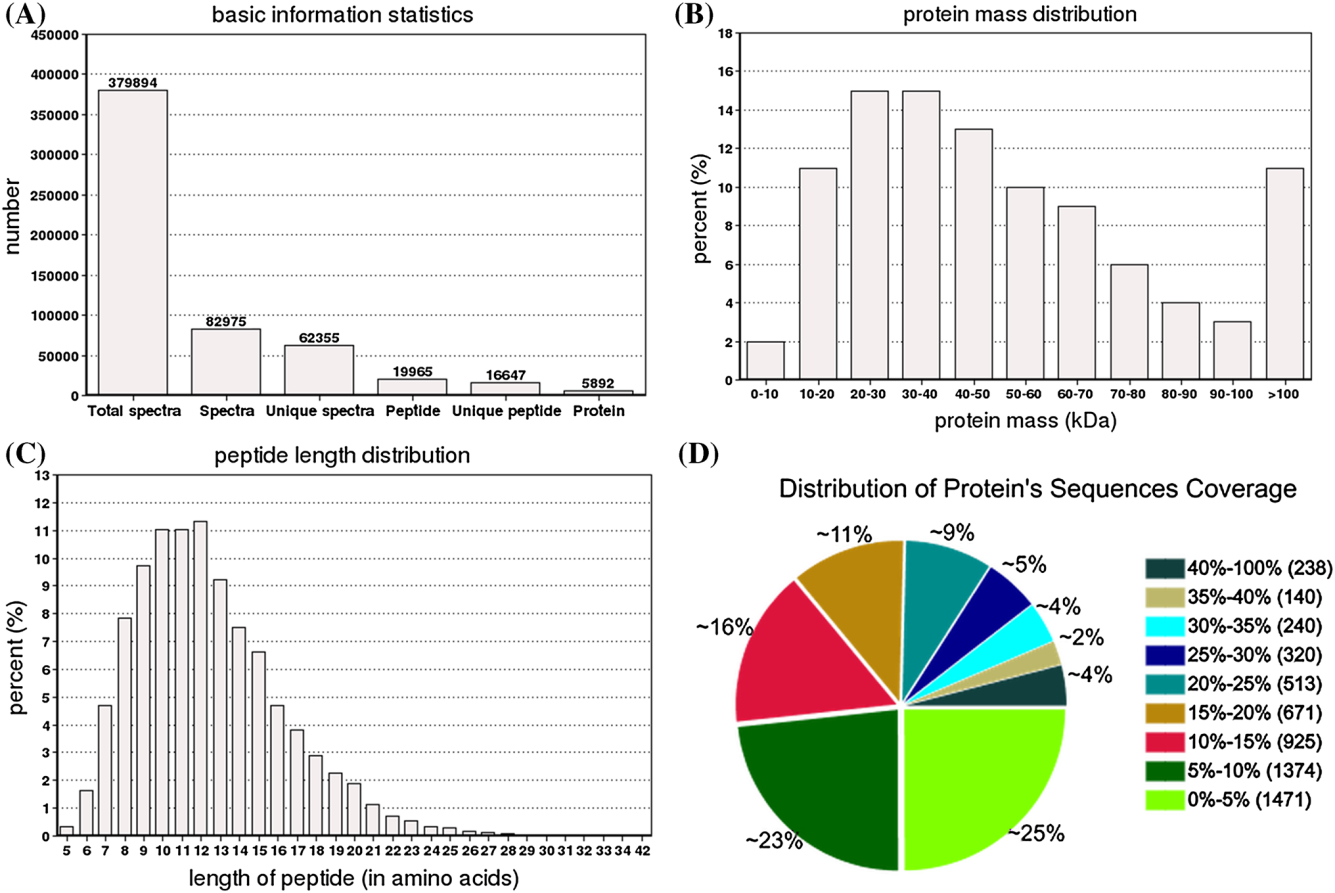
Protein identification by iTRAQ. **a** Number of spectra, peptide and protein; **b** percentage of protein mass distribution; **c** peptide length distribution; **d** distribution of protein’s sequence coverage

iTRAQ based comparative proteome was employed to assess protein changes amongst NEC, EC and SEs in cotton. In the three samples, a total of 379,894 (62,335 unique) spectra were obtained. Of these spectra, 19,965 were identified as peptides (16,647 unique peptides) and 5892 as proteins (Fig. 2a). The mass of the identified proteins showed a normal distribution, with 0–50, 51–100 and > 100 kDa proteins accounting for 56, 32 and 11%, respectively (Fig. 2b). The peptide number distribution of the proteins (number of amino acids) with 1–5 peptides, 6–10 peptides, and 11 or more peptides comprised 5160, 569, and 163, respectively (Fig. 2c), most peptides have 9–13 amino acids, very few peptides have 5 or less amino acids. The distribution of protein sequence coverage with 40–100, 30–40, 20–30, 10–20, and under 10% variations accounted for 4.04, 6.45, 14.14, 27.09 and 48.29%, respectively (Fig. 2d).

### Functional categories of the proteins in GO and COG

**Fig. 3:**
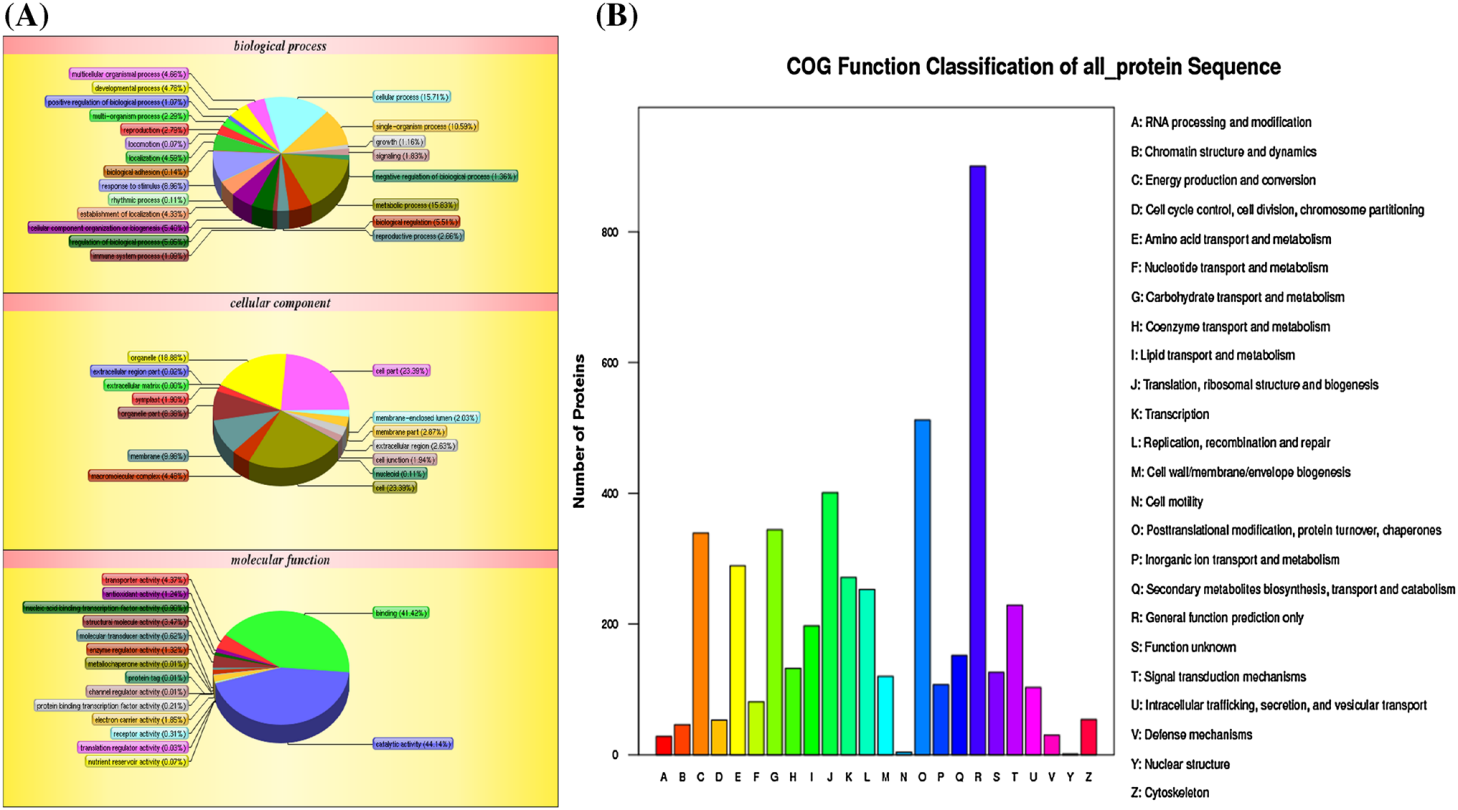
GO and COG analysis of identified proteins in somatic embryogenesis. **a** GO analysis of DAPs; **b** COG analysis of DAPs

GO analysis was applied to annotate the 5892 proteins identified in NEC, EC and SEs. At the biological process level, proteins involved in metabolic process, cellular process, single-organism process and response to stimulus process accounted for 15.83, 15.71, 10.59 and 8.96%, respectively, and other proteins accounted for about 50%. At the cellular component level, proteins related to cell, cell part, organelle, membrane and organelle part accounted for 23.39, 23.39, 18.88, 9.98 and 8.38%, respectively, and others accounted for 14.38%. At the molecular function level, proteins with catalytic activity, binding activity, transporter activity and structural molecular activity covered 44.14, 41.42, 4.37, and 3.47%, respectively, and others covered 6.6% (Fig. 3a). Analysis of the identified proteins by COG suggested that general function prediction, posttranslational modification\protein turnover\chaperones, translation\ribosomal structure and biogenesis, carbohydrate transport and metabolism, energy production and conversion were the top five types of functions (Fig. 3b).

### Identification of differentially accumulated proteins

**Fig. 4:**
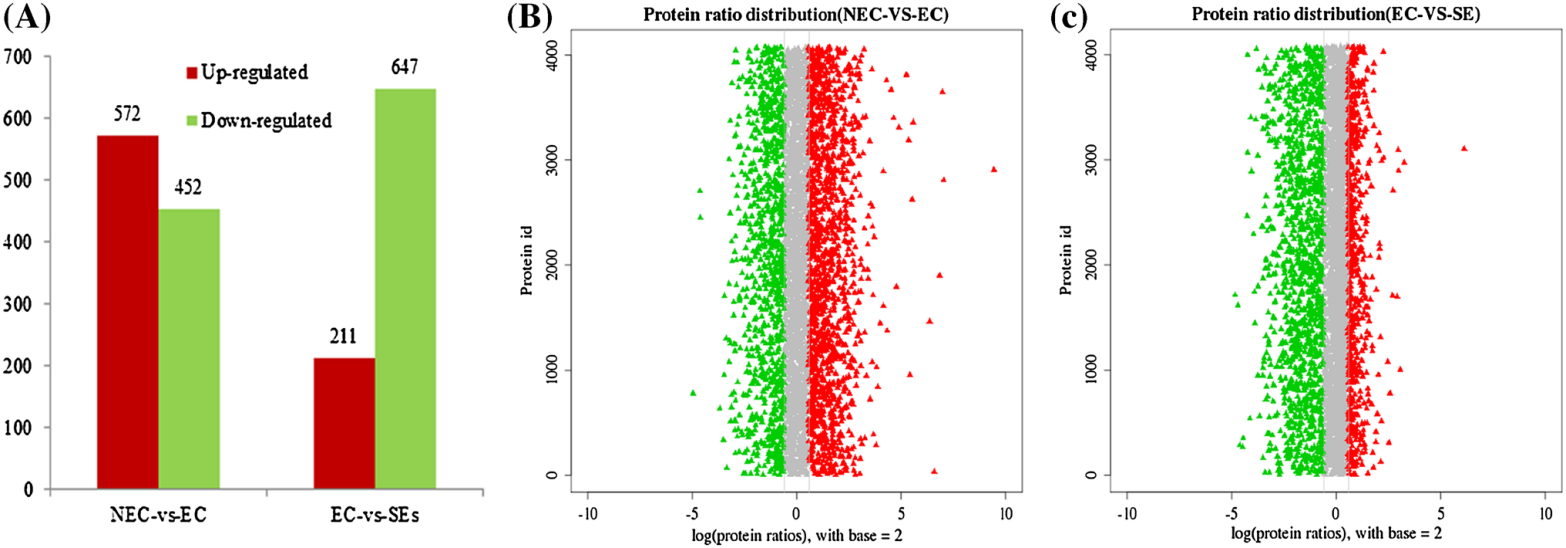
Distribution of DAPs. **a** Number of up-and down-regulated DAPs in NEC vs. EC and EC vs. SEs; **b** and **c** protein ratio distribution in NEC vs. EC and EC vs. SEs, respectively

Differentially accumulated proteins (DAPs) were defined as those with a > 1.5-fold change in relative abundance (P < 0.05) between NEC and EC, or between EC and SEs. In total, 1024 and 858 DAPs were identified in NEC versus EC, and EC versus SEs, respectively. Of the 1024 DAPs identified between NEC and EC, 452 and 572 proteins were down- and up-regulated in EC, respectively. Of the 858 DAPs identified between EC and SEs, 647 and 221 proteins were down- and up-regulated in SEs, respectively (Fig. 4a). Protein ratio distribution was focused in general function prediction, posttranslational modification\protein turnover\chaperones (Fig. 4b, c).

**Fig. 5:**
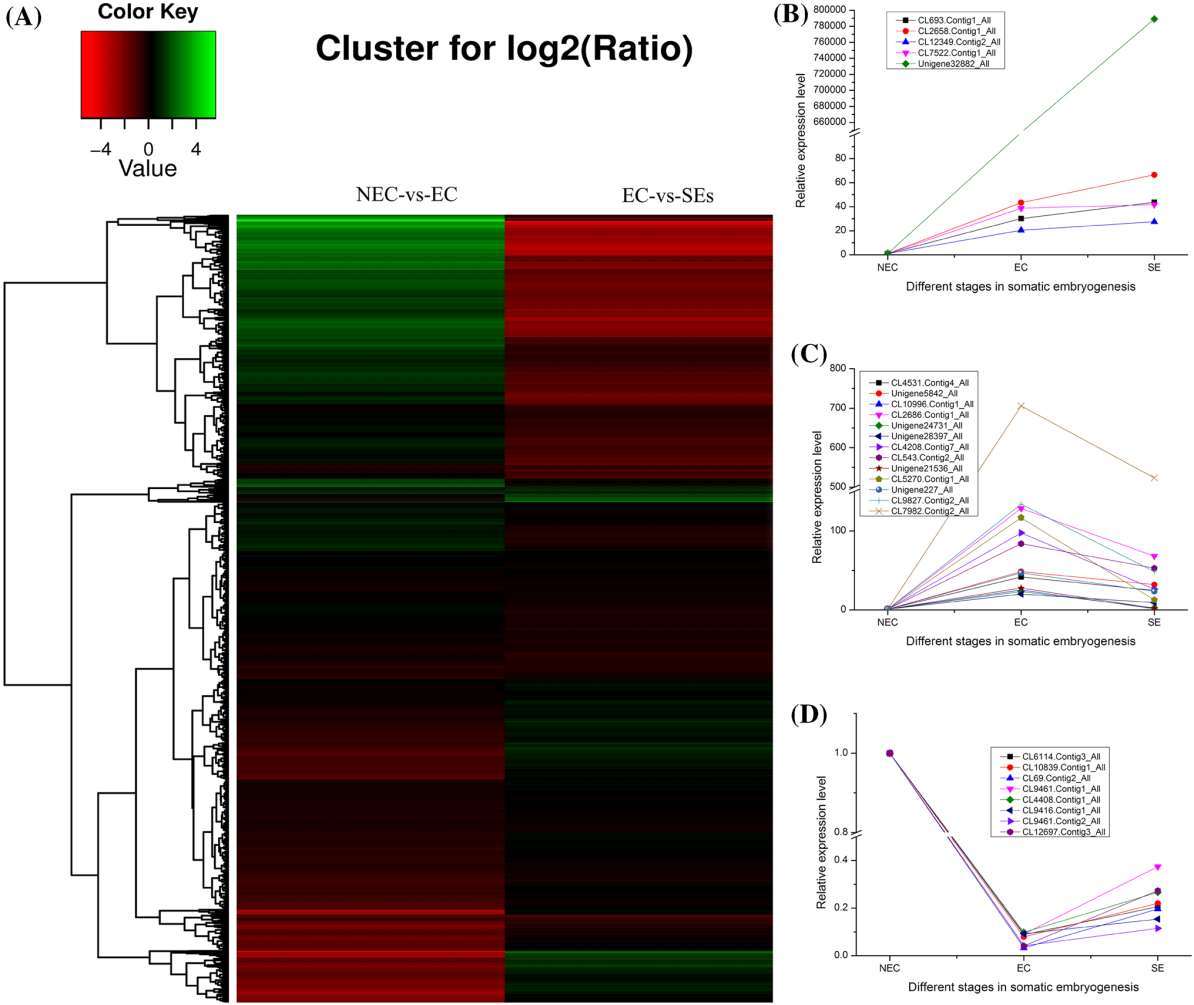
Cluster analysis of DAPs in NEC, EC and SEs. **a** Cluster analysis of DAPs in NEC, EC and SEs; **b** representative DAPs showing continuous accumulation from NEC to EC, and from EC to SEs, i.e. with a pattern of up- to up-regulation; **c** representative DAPs showing accumulation from NEC to EC, but decrease from EC to SEs, i.e. with a pattern of up- to down-regulation; **d** representative DAPs showing decrease from NEC to EC, but increase from EC to SEs, i.e. with a pattern of down- to up-regulation

To investigate the overall dynamics of proteome changes in SEM of Xinluzao 33, we performed a hierarchical cluster analysis for the DAPs identified in NEC, EC and SEs. This analysis suggested three types of protein expression patterns, i.e. up- to up-regulation, up- to down-regulation, and, down- to up-regulation (Fig. 5).

### Pathway annotation of DAPs in KEGG

The 1882 DAPs identified were classified into 127 families based on KEGG. About 48% of them were classified into metabolic pathways and biosynthesis of secondary metabolites, which suggests that metabolites were remarkably changed during SEM. Furthermore, about 2–5% of DAPs were involved in pathways related to ribosome, spliceosome, protein processing in endoplasmic reticulum, RNA transport, glycolysis/gluconeogenesis, plant hormone signal transduction, plant-pathogen interaction, mRNA surveillance pathway, starch and sucrose metabolism, purine metabolism, pyruvate metabolism, phenylpropanoid biosynthesis, amino sugar and nucleotide sugar metabolism and oxidative phosphorylation. The aforementioned pathways covered more than 90% of the DAPs. Ribosome, spliceosome, protein processing in endoplasmic reticulum and RNA transport are involved in genetic information transcription and processing, which suggests that remarkable changes of genetic information occurred in SEM. We listed the top 25 pathways based on the number of DAPs in Table 2.

**Table 2:** Pathway annotation of differentially accumulated proteins in somatic embryogenesis

No.	Pathway	DAPs (4172)	
1	Metabolic pathways	1270	30.44%
2	Biosynthesis of secondary metabolites	736	17.64%
3	Ribosome	199	4.77%
4	Spliceosome	174	4.17%
5	Protein processing in endoplasmic reticulum	168	4.03%
6	RNA transport	167	4.00%
7	Glycolysis/gluconeogenesis	129	3.09%
8	Plant hormone signal transduction	121	2.90%
9	Plant-pathogen interaction	120	2.88%
10	mRNA surveillance pathway	111	2.66%
11	Starch and sucrose metabolism	110	2.64%
12	Purine metabolism	108	2.59%
13	Pyruvate metabolism	107	2.56%
14	Phenylpropanoid biosynthesis	100	2.40%
15	Amino sugar and nucleotide sugar metabolism	95	2.28%
16	Oxidative phosphorylation	90	2.16%
17	Pyrimidine metabolism	82	1.97%
18	RNA degradation	81	1.94%
19	Endocytosis	75	1.80%
20	Carbon fixation in photosynthetic organisms	74	1.77%
21	Glutathione metabolism	71	1.7%
22	Peroxisome	60	1.44%
23	Ascorbate and aldarate metabolism	57	1.37%
24	Proteasome	56	1.34%
25	Arginine and proline metabolism	53	1.27%

### Transcriptional analysis of candidate genes encoding DAPs

**Fig. 6:**
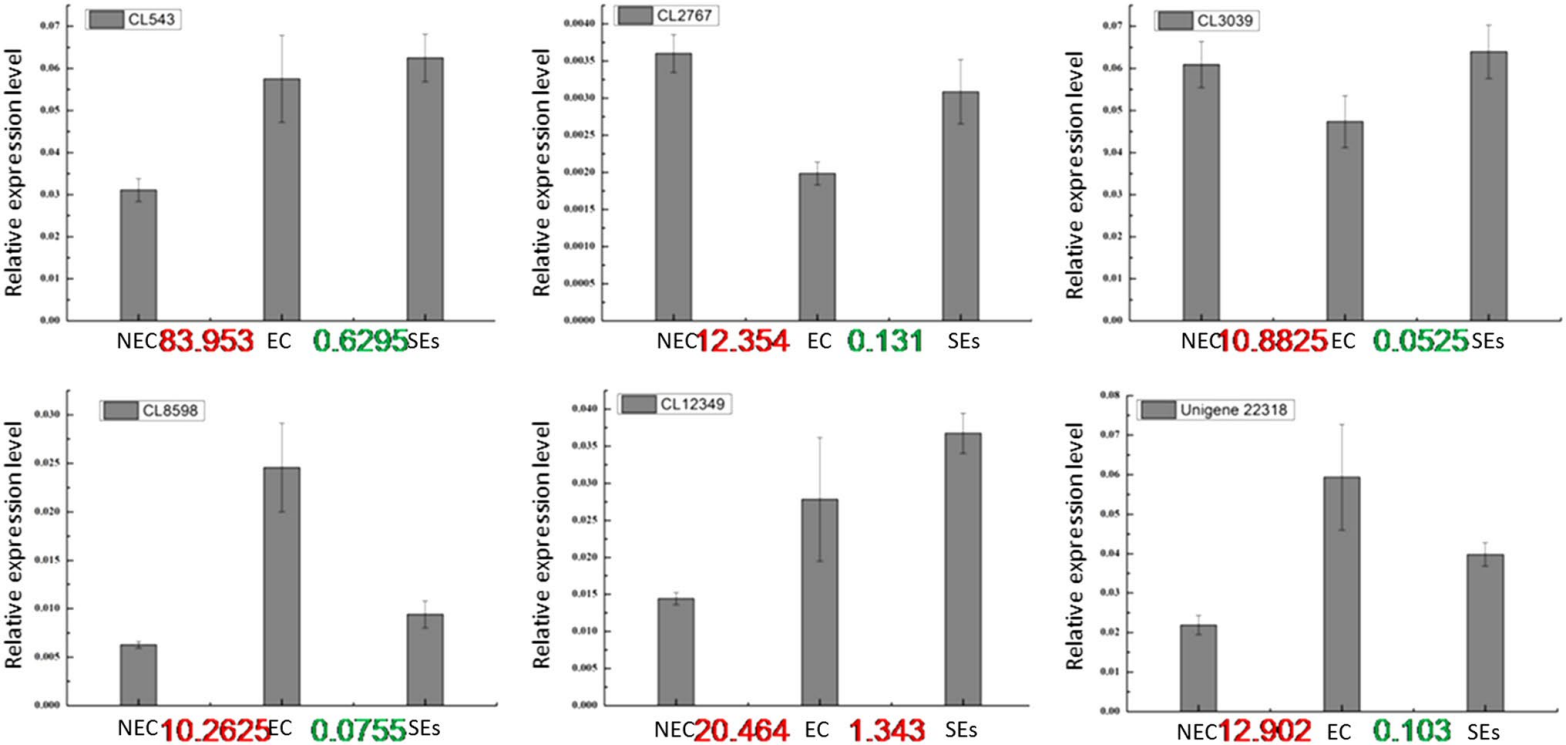
Transcriptional analysis of candidate genes encoding for the DAPs the numbers showing on the X-axis are the fold changes of the accumulated level of the corresponding DAP in EC versus NEC and SEs versus EC based on the iTRAQ data. Red and green denote up and down regulation, respectively

To know the correlation between the expression levels of DAPs and their corresponding genes, we selected six DAPs and analyzed their transcript levels in NEC, EC and SE. The six selected genes were CL543.Contig2_All (tubulin beta-1), CL12349.Contig2_All (conserved hypothetical protein), CL8598.Contig1_All (rRNA-processing protein fcf2-like isoform 1), CL3039.Contig1_All (serine/arginine-rich splicing factor RS2Z32-like isoform 1), CL2767.Contig2_All (uncharacterized protein At2g39795), and Unigene22318_All (serine/arginine rich splicing factor). Three genes, including CL8598.Contig1_All, CL12349.Contig2_All and Unigene22318_All, showed good correlation between the expression levels of protein and mRNA. For the other three DAPs, no good correlation between their expression levels of protein and mRNA was observed, suggesting that their protein levels might be determined not only at the transcript level but also at the post-translational level (Fig. 6).

### SERK, LEC and CKI proteins are differentially accumulated during cotton SEM

The *SERK* family genes and *LEC1* (*LEAFY COTYLEDON1*) were implicated in the signal transduction pathway and transcriptional regulation during SEM (Zuo et al. 2002; Gaj et al. 2005; aan den Toorn et al. 2015; Podio et al. 2014). *LEC1, LEC2* and *FUS3* are the key genes controlling the SEM process in plants (Gaj et al. 2005; Fambrini et al. 2006). The SEM capacity was completely repressed in double (*lec1 lec2, lec1 fus3*, or *lec2 fus3*) and triple (*lec1 lec2 fus3*) *Arabidopsis thaliana* mutants (Gaj et al. 2005). The expression level of *LEC2*/*FUS* changed rapidly in response to auxin treatment (Stone et al. 2008), suggesting that *LEC*/*FUS* may be the upstream genes in the auxin signaling pathway (Gaj et al. 2005; Stone et al. 2001). According to our proteomic profile, these SEM related proteins were differentially accumulated during cotton SEM (Table 3). Both SERK and LEC proteins were up-regulated in EC and SEs compared with NEC. These two types of proteins are multifunctional regulators in both zygotic and SEM with a role in hormone signaling and stress responses (Mahdavi-Darvari et al. 2015), and have been used as markers of totipotency in plant species. CASEIN KINASE I, which have been previously demonstrated to play a role in SEM of cotton and other plants, was down-regulated in EC and SEs compared with NEC (Table 3), consistent with the previously published result in cotton (Mahdavi-Darvari et al. 2015). GhCKI is a negative regulator of cotton SEM by regulating auxin homeostasis through the LEC1-CKI-TCP15-PIF4 network (Min et al. 2015). Our data thus confirmed the possible role of SERK, LEC and CKI during cotton SEM.

**Table 3:** Fold changes of selected DAPs in NEC vs. EC and EC vs. SEs

No.	Protein ID	Protein name	Pathway annotation in KEGG	NEC vs. EC	EC vs. SEs
1	CL2809.Contig4_All	GH3	Auxin signal	− 1.8	0.6
2	CL2472.Contig1_All	CRE1	CTK signal	2.7	− 3.1
3	Unigene20442_All	AHP	CTK signal	–	0.8
4	CL11792.Contig4_All	B-ARR	CTK signal	1.8	− 1.6
5	Unigene12438_All	DELLA	Gibberellin signal	−	− 1.1
6	Unigene13500_All	GID	Gibberellin signal	1.8	–
7	CL1027.Contig3_All	ABF	ABA signal	2.7	− 2
8	CL5280.Contig2_All	PYR/PYL	ABA signal	–	1.5
9	CL7181.Contig7_All	CTR1	Ethylene signal	0.8	− 0.9
10	CL4433.Contig1_All	MPK6	Ethylene signal	− 0.7	–
11	CL12267.Contig1_All	BAK1	Brassinosteroid signal	− 1.6	− 2.6
12	CL10648.Contig2_All	BRI1	Brassinosteroid signal	− 0.8	− 2.3
13	CL7395.Contig2_All	TCH4	Brassinosteroid signal	–	0.8
14	Unigene11483_All	PR1	SA signal	− 2.8	–
15	CL12586.Contig1_All	SERK	Somatic embryogenesis related proteins	4.8	3.7
16	CL7993.Contig1_All	LEC	Somatic embryogenesis related proteins	2.2	1.7
17	CL4025.Contig1_All	CKI	Somatic embryogenesis related proteins	− 1.4	− 1.1
18	CL11109.Contig1_All	ADC	Polyamines	2.9	3.7
19	Unigene7615_All	SAMDC	Polyamines	3.1	2.8
20	CL1916.Contig10_All	SPMS	Polyamines	1.7	2.3
21	CL2833.Contig2_All	CAT	Peroxisome	1.7	− 2.1
22	CL517.Contig2_All	SOD	Peroxisome	− 1.6	− 2.2
23	CL600.Contig1_All	FabF	Fatty acid	2.5	–
24	CL1721.Contig2_All	FatA	Fatty acid	1.4	–
25	Unigene19300_All	FabG	Fatty acid	–	1.8
26	Unigene7832_All	FabZ	Fatty acid	–	0.7
27	Unigene9530_All	SERK	Transcription factor	1.8	2.4
28	CL11093.Contig1_All	LEC1	Transcription factor	1.7	3.1
29	Unigene10107_All	WUS	Transcription factor	4.1	4.1
30	CL7829.Contig6_All	CKI	Transcription factor	2.8	− 1.9
31	CL11268.Contig2_All	ND1	Oxidative phosphorylation	3	1
32	CL4559.Contig2_All	ND6	Oxidative phosphorylation	8.2	3.7
33	CL3850.Contig1_All	COX3	Oxidative phosphorylation	14.4	12.9
34	CL4756.Contig1_All	COX15	Oxidative phosphorylation	1.2	1.4
36	CL7474.Contig1_All	*CDPK*	Stress-response	2.9	− 3.2
37	CL10919.Contig1_All	*HSP90*	Stress-response	6.8	− 1.3
38	Unigene15666_All	*SGT1*	Stress-response	2.2	2.3
39	CL4692.Contig1_All	4CL	Secondary metabolites	− 1.9	2.4

### Plant hormone signal transduction involved in cotton somatic embryogenesis

Plant hormones played important roles in SEM either working alone or through crosstalk with each other (Yang and Zhang 2010; Yang et al. 2012; Xu et al. 2013; Cheng et al. 2016). Based on our proteome profile, a number of proteins related to hormone (including auxin, cytokinine, gibberellin, ABA, ethylene, brassinosteroid and SA) metabolism and signaling pathways were differentially expressed in EC and/or SEs.

The regulatory role of these proteins was probably achieved by establishing auxin and cytokinine gradients during the induction phase of SEM. They are essential for initiating dedifferentiation and cell division of already differentiated cells before they could express embryogenic competence. Despite the absolute requirement for exogenous auxin to sustain growth of plant cells cultured in vitro, cultured plant cells produce substantial amounts of the native auxin, IAA. Comprehensive studies have been conducted on callogenesis from several cotton cultivars using various explants, and different combinations of growth regulators. We found that GH3, an auxin-induced protein involved in the auxin signal pathway, was differentially expressed in NEC, EC and SEs (Table 3). GH3 was down-regulated in EC but up-regulated in SE, consistent with the result of low concentration of auxin promoting EC differentiation in cotton (Sun et al. 2006; Zhou et al. 2016).

Cytokinine induces SEM and plant regeneration in many plant species (Wang and Chong 2015). Proteins involved in the cytokinine signal pathway, such as CRE1 (a cytokinin receptor), AHP and B-ARR, showed a differentially expressed pattern in EC and/or SEs. Compared with NEC, EC had an up-regulated level of both CRE1 and B-ARR, and compared with EC, SEs had a down-regulated level of both CRE1 and B-ARR. AHP was unchanged between NEC and EC but slightly up-regulated in SEs (Table 3).

In the gibberellin signal pathway, GID1 and DELLA showed differential and opposite expression pattern in NEC versus EC and EC versus SEs. From NEC to EC, GID1 was up-regulated and then remained unchanged in SEs. In contrast, from NEC to EC, the level of DELLA was unchanged but was then down-regulated in SEs.

Abscisic acid plays an important role in the accumulation of nutritive products during the development and maturation of asexual embryo (Jin et al. 2014). PYR/PYL and ABF, involved in the ABA signal pathway, showed differential expression pattern during cotton somatic embryogenesis. From NEC to SEs, ABF was up-regulated in EC and then down-regulated in SEs. The level of PYR/PYL showed no significant difference between NEC and EC, and was up-regulated in SEs compared to EC.

The role of ethylene on embryogenic induction is complicated by its inconsistent effects in different plants and culture systems. In our study, CTR1, which is involved in the ethylene signal pathway, was slightly down-regulated in SEs compared to EC, suggesting a possible negative role of CTR1 in cotton SEM.

Brassinosteroids (BRs) are growth-promoting steroid hormones that occur widely across the plant kingdom and regulate multiple aspects of physiological responses essential for both vegetative and reproductive development (Clouse 2011). Proteins like BAK1 and BRI1, involved in the brassinosteroid signal pathway, were mostly down-regulated in both NEC versus EC and EC versus SEs.

PR-1, involved in the salicylic acid signal pathway, was down-regulated in EC compared to NEC and then remained unchanged in SEs. These results of hormone signaling related proteins demonstrated an essential role of hormones and their complicated crosstalk in cotton SEM.

### Polyamines involved in cotton somatic embryogenesis

Polyamines have been shown to be involved in many physiological processes including regulation of cell division, senescence, responses to both biotic and abiotic stress (Marco et al. 2011; Minocha et al. 2014). Based on KEGG analysis and GO annotation, proteins involved in polyamine synthesis or metabolism, including SAMDC, ADC and SPMS, were all differentially expressed during cotton SEM. All of these proteins showed a higher expression level in EC compared with NEC and continued to increase in SEs (Table 3; Fig. 5), consistent with our recent transcriptome results showing that polyamines could promote cotton SEM (Cheng et al. 2015, 2016). Both ADC mRNA and protein were localized in dividing cells of embryo meristems, probably required for mitosis (Vuosku et al. 2006). Exogenous application of Put and Spm enhanced the growth of embryogenic cultures of *Araucaria angustifolia* and significantly affected the endogenous concentrations of PAs, IAA and ABA in embryogenic tissues (Steiner et al. 2007). Our previous results also showed that exogenous application of polyamines enhanced the growth and development of cotton SEs, which could be achieved by PAs-mediated regulation of specific physiological processes important for cellular differentiation (Cheng et al. 2016).

### Cotton somatic embryogenesis regulated by reactive oxygen species (ROS)

The NADH dehydrogenase related proteins, ND1 and ND6, were up-regulated in EC and SEs compared to NEC. The cytochrome C oxidases, including COX3 and COX15, showed an up-regulated pattern in EC and SEs. The ATPases (includes A, F and V-type) had a relatively high expression level in EC (Table 3). Differential expression of proteins related to oxidative phosphorylation indicated that energy metabolism was very active in the transition from NEC to EC and development of SEs.

Additionally, proteins involved in the protective system, including CAT and SOD, were up-regulated in EC and SEs (Table 3). The antioxidant system might play a role in maintaining ROS homeostasis during the differentiation and development of EC and SEs. These results suggested that ROS played a crucial role in the development of EC and SEs during cotton SEM.

The stress response related proteins, such as CDPK, HSP90 and SGT, were also differentially expressed during cotton SEM (Table 3), suggesting that stresses might be involved in regulating EC proliferation and differentiation of SEs.

These results indicated that ROS was closely related to cotton somatic embryogenesis, and as signal molecules, the role of ROS in regulating SE was unclear. A recent research reported that the ROS homeostasis might regulate somatic embryogenesis via the regulation of auxin signaling in Cotton (Zhou et al. 2016).

### Fatty acid biosynthesis and metabolism in cotton somatic embryogenesis

The composition of fatty acid rapidly changed during somatic embryo development following the induction of embryogenesis in *D. carota* (Warren and Fowler 1979; Makarenko et al. 2016). In our iTRAQ profile, many fatty acid biosynthesis and metabolism related proteins, such as FabH, accC, FatB and FabD, were differentially accumulated. In the fatty acid biosynthesis pathway, FabH, accC, FatB and FabD were up-regulated in both EC and SEs (Table 3). A previous study reported that ectopic expression of *LEC2* induces accumulation of fatty acid during the maturation phase of embryogenesis (Stone et al. 2008). These results indicated that fatty acid might play an important role in cotton SEM.

### Genetic information rapidly changed during somatic embryogenesis

Cellular dedifferentiation and redifferentiation are the two important events during plant regeneration via SEM (Yang and Zhang 2010; Zimmerman 1993). In this study, we found that many DAPs are directly or indirectly involved in genetic information processing, including transcription, post-transcription, translation and post-translation. Five of the top 10 pathways of DAPs (including 19.63% of all DAPs) were annotated as related to genetic information processing pathways. These DAPs exhibited opposite expression pattern in NEC *vs* EC and EC versus SE, i.e. down-regulated in EC formation and up-regulated in SE differentiation (data not shown). These results suggested that some structural genes might be down-regulated to save energy during cellular dedifferentiation and subsequently were alternatively expressed during cellular redifferentiation through rapid genetic information processing (Noah et al. 2013).

### Secondary metabolites broadly involved in somatic embryogenesis

Secondary metabolites, including terpene, phenolic and nitrogen-containing compound, are widely involved in growth, development and defense and play important roles in plant life cycle (Bartwal et al. 2013; Chacón et al. 2013). According to the KEGG pathway annotation, 17.64% of all DAPs were found to be involved in biosynthesis of secondary metabolites. Actually, plant tissue culture process was accompanied by a large number of metabolism and transport. Although the precise function of a certain secondary metabolite was still unclear, our findings provided foundation for further studies. For example, we found that 4CL (4-coumarate-CoA-ligase), one of the key enzymes involved in phenylpropanoid metabolism and lignin biosynthesis, was reversely expressed during differentiation of EC and SE, suggesting that lignin and related metabolites might function differently during EC formation and SE development.

### Correlation analysis of transcriptome and proteome data

**Fig. 7:**
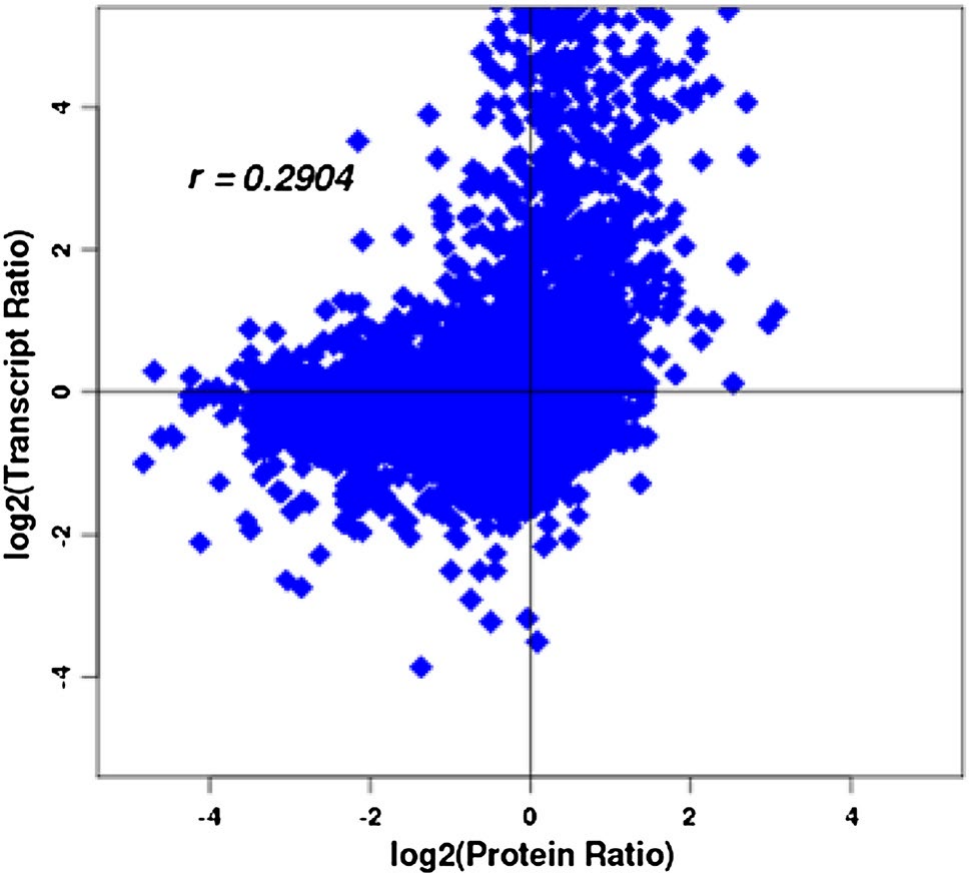
Correlation of changes of protein accumulation and gene expression in NEC versus EC

To better understand the relationship and interplay between transcriptome and proteome in cotton SEM, we did correlation analysis using the transcriptome data we previously generated from the same samples (Cheng et al. 2016) and the proteome data generated in this study. The analysis was performed at three different levels, i.e. based on the number of proteins and transcripts identified (Identification), the number of proteins and transcripts that could be quantified (Quantification) and the number of proteins and transcripts differentially expressed (Differential expression) (Table 4). The number of identified proteins was based on the iTRAQ qualitative screen results. The proteins represented by two or more peptides were considered as quantifiable, for which the peptide ratio was calculated, and the median peptide ratio was used to represent the corresponding protein level. The criteria of differentially expressed proteins have been mentioned above. The P value for differentially expressed genes was 0.001. As a result, almost all identified and quantifiable proteins (> 99%) had a corresponding transcript, but only 18.6% (160/858), 56.3% (576/1024) and 53.9% (263/488) of DAPs had a corresponding differentially expressed transcript in EC vs. SEs, NEC vs. EC and NEC vs. SEs, respectively (Table 4). These results indicate that the presence of a protein was well supported by the presence of a corresponding transcript, but the correlation between the expression level or change of a protein and its corresponding transcript is poor and seems to be tissue and developmental stage dependent. This was further supported by the low correlation coefficient between the changes of proteins and their corresponding transcripts in the three samples. For example, the correlation coefficient between the changes of protein and its corresponding transcript in NEC vs. EC was 0.29 (Fig. 7). These results suggest that analysis of both transcriptome and proteome is required to identify key players critical for cotton SEM. To support this notion, the gene expression levels of about a third of the DAPs listed in Table 4 were also significantly different in the three developmental stages (Jiménez and Thomas 2006), and these genes had a good correlation between the changes of transcript and protein.

**Table 4:** Correlation analyses of transcripts and proteins

Comparison group	Level	Number of proteins	Number of transcripts	Number of proteins with a corresponding transcript
EC vs. SEs	Identification	5892	87,790	5831
EC vs. SEs	Quantitation	4080	87,790	4061
EC vs. SEs	Differential expression	858	11,888	160
NEC vs. EC	Identification	5892	100,750	5885
NEC vs. EC	Quantitation	4077	100,750	4075
NEC vs. EC	Differential expression	1024	42,532	576
NEC vs. SEs	Identification	5892	100,509	5883
NEC vs. SEs	Quantitation	4086	100,509	4082
NEC vs. SEs	Differential expression	488	29,408	263

## Conclusion

In conclusion, our proteomics analysis unveiled the complex and dynamic nature of the protein network involved in cell dedifferentiation and redifferentiation during cotton SEM, and identified the key regulators for cotton SEM. Our proteomics results confirmed our previous transcriptomic results on the role of the polyamine metabolic pathways and stress responses in cotton SEM. We propose that SEM related proteins are the top layer players regulating reprogramming of the transcriptome required for cell dedifferentiation and redifferentiation, and that the hormone synthesis and signaling pathways, the stress-response pathways and the polyamine metabolic pathways function side-by-side and synergistically to promote embryogenic cell initiation, somatic embryogenesis and plantlet regeneration. The possible role of certain genes/proteins and pathways regulating cotton SEM will be further investigated in our future work.
